# Supplementary material for: An open-source framework for large-scale, flexible evaluation of biomedical text mining systems
Source: J Biomed Discov Collab. 2008 Jan 29;3:1. doi: 10.1186/1747-5333-3-1 (PMC2276192; doi:10.1186/1747-5333-3-1)
Supplement: Additional file 1 — Evaluation platform source code. The additional file contains the software evaluation framework discussed in this paper. The most current release can be downloaded from SourceForge.net [1]. See the accompanying README file for instructions on installation and use. Also included in the distribution is a collection of UIMA wrappers for some commonly used BioNLP tools and annotated corpora. [file 1747-5333-3-1-S1.zip › bionlp_uima_repository-1.0.1/docs/api/edu/uchsc/ccp/uima/annotation/CCPDocumentInformation.html]

CCPDocumentInformation (Center for Computational Pharmacology Util/UIMA API)


|  |  |  |
| --- | --- | --- |
| **Overview** \| **Package** \| **Class** \| **Tree** \| **Deprecated** \| **Index** \| **Help** | |  |
| **PREV CLASS**   **NEXT CLASS** | **FRAMES**    **NO FRAMES**     **All Classes** |  |
| SUMMARY: NESTED \| FIELD \| CONSTR \| METHOD | DETAIL: FIELD \| CONSTR \| METHOD |  |


---


## edu.uchsc.ccp.uima.annotation Class CCPDocumentInformation

```
java.lang.Object
  org.apache.uima.cas.impl.FeatureStructureImpl
      org.apache.uima.jcas.cas.TOP
          org.apache.uima.jcas.cas.AnnotationBase
              org.apache.uima.jcas.tcas.Annotation
                  edu.uchsc.ccp.uima.annotation.CCPAnnotation
                      edu.uchsc.ccp.uima.annotation.CCPDocumentAnnotation
                          edu.uchsc.ccp.uima.annotation.CCPDocumentInformation
```

**All Implemented Interfaces:**: Cloneable, org.apache.uima.cas.AnnotationBaseFS, org.apache.uima.cas.FeatureStructure, org.apache.uima.cas.text.AnnotationFS

---

``` public class CCPDocumentInformation extends CCPDocumentAnnotation ```

The CCPDocumentInformation annotation includes document metadata such as the document ID, document collection ID, secondary document IDs, document size, etc.
Updated by JCasGen Tue Aug 28 16:44:10 MDT 2007
XML source: /home/williamb/eclipse/RELEASED\_SOURCE\_CODE/descriptors/typeSystem/CCPTypeSystem.xml

---

| **Field Summary** | |
| --- | --- |
| `static int` | `type` |
| `static int` | `typeIndexID` |

**Fields inherited from class org.apache.uima.jcas.cas.TOP**

`addr, jcasType`


| **Constructor Summary** | |
| --- | --- |
| `protected` | `CCPDocumentInformation()`             Never called. |
|  | `CCPDocumentInformation(int addr, org.apache.uima.jcas.cas.TOP_Type type)`             Internal - constructor used by generator |
|  | `CCPDocumentInformation(org.apache.uima.jcas.JCas jcas)` |
|  | `CCPDocumentInformation(org.apache.uima.jcas.JCas jcas, int begin, int end)` |


| **Method Summary** | |
| --- | --- |
| `String` | `getClassificationType()`             getter for classificationType - gets This String provides a means for classifying a particular document. |
| `int` | `getDocumentCollectionID()`             getter for documentCollectionID - gets The document collection ID is an Integer that uniquely identifies a particular document collection. |
| `String` | `getDocumentID()`             getter for documentID - gets The document ID is a String representing a unique identifier for a particular document within a particular document collection. |
| `int` | `getDocumentSize()`             getter for documentSize - gets The size of a document is logged as the number of characters it contains. |
| `org.apache.uima.jcas.cas.StringArray` | `getSecondaryDocumentIDs()`             getter for secondaryDocumentIDs - gets This StringArray is used for secondary document ID storage. |
| `String` | `getSecondaryDocumentIDs(int i)`             indexed getter for secondaryDocumentIDs - gets an indexed value - This StringArray is used for secondary document ID storage. |
| `int` | `getTypeIndexID()` |
| `void` | `setClassificationType(String v)`             setter for classificationType - sets This String provides a means for classifying a particular document. |
| `void` | `setDocumentCollectionID(int v)`             setter for documentCollectionID - sets The document collection ID is an Integer that uniquely identifies a particular document collection. |
| `void` | `setDocumentID(String v)`             setter for documentID - sets The document ID is a String representing a unique identifier for a particular document within a particular document collection. |
| `void` | `setDocumentSize(int v)`             setter for documentSize - sets The size of a document is logged as the number of characters it contains. |
| `void` | `setSecondaryDocumentIDs(int i, String v)`             indexed setter for secondaryDocumentIDs - sets an indexed value - This StringArray is used for secondary document ID storage. |
| `void` | `setSecondaryDocumentIDs(org.apache.uima.jcas.cas.StringArray v)`             setter for secondaryDocumentIDs - sets This StringArray is used for secondary document ID storage. |

**Methods inherited from class org.apache.uima.jcas.tcas.Annotation**

`getBegin, getCoveredText, getEnd, getStart, setBegin, setEnd`

**Methods inherited from class org.apache.uima.jcas.cas.AnnotationBase**

`getSofa, getView`

**Methods inherited from class org.apache.uima.jcas.cas.TOP**

`addToIndexes, addToIndexes, equals, getAddress, getCAS, getCASImpl, getLowLevelCas, hashCode, removeFromIndexes, removeFromIndexes`

**Methods inherited from class org.apache.uima.cas.impl.FeatureStructureImpl**

`clone, getBooleanValue, getByteValue, getDoubleValue, getFeatureValue, getFeatureValueAsString, getFloatValue, getIntValue, getLongValue, getShortValue, getStringValue, getType, prettyPrint, prettyPrint, prettyPrint, setBooleanValue, setByteValue, setDoubleValue, setFeatureValue, setFeatureValueFromString, setFloatValue, setIntValue, setLongValue, setShortValue, setStringValue, toString, toString`

**Methods inherited from class java.lang.Object**

`finalize, getClass, notify, notifyAll, wait, wait, wait`

**Methods inherited from interface org.apache.uima.cas.AnnotationBaseFS**

`getView`

**Methods inherited from interface org.apache.uima.cas.FeatureStructure**

`clone, equals, getBooleanValue, getByteValue, getCAS, getDoubleValue, getFeatureValue, getFeatureValueAsString, getFloatValue, getIntValue, getLongValue, getShortValue, getStringValue, getType, hashCode, setBooleanValue, setByteValue, setDoubleValue, setFeatureValue, setFeatureValueFromString, setFloatValue, setIntValue, setLongValue, setShortValue, setStringValue`

**Field Detail**

### typeIndexID

```
public static final int typeIndexID
```

---


### type

```
public static final int type
```


**Constructor Detail**

### CCPDocumentInformation

```
protected CCPDocumentInformation()
```

:   Never called. Disable default constructor

---


### CCPDocumentInformation

```
public CCPDocumentInformation(int addr,
                              org.apache.uima.jcas.cas.TOP_Type type)
```

:   Internal - constructor used by generator

---


### CCPDocumentInformation

```
public CCPDocumentInformation(org.apache.uima.jcas.JCas jcas)
```

---


### CCPDocumentInformation

```
public CCPDocumentInformation(org.apache.uima.jcas.JCas jcas,
                              int begin,
                              int end)
```


**Method Detail**

### getTypeIndexID

```
public int getTypeIndexID()
```

:   **Overrides:**: `getTypeIndexID` in class `CCPDocumentAnnotation`

---


### getDocumentID

```
public String getDocumentID()
```

:   getter for documentID - gets The document ID is a String representing a unique identifier for a particular document within a particular document collection.

---


### setDocumentID

```
public void setDocumentID(String v)
```

:   setter for documentID - sets The document ID is a String representing a unique identifier for a particular document within a particular document collection.

---


### getDocumentCollectionID

```
public int getDocumentCollectionID()
```

:   getter for documentCollectionID - gets The document collection ID is an Integer that uniquely identifies a particular document collection.

---


### setDocumentCollectionID

```
public void setDocumentCollectionID(int v)
```

:   setter for documentCollectionID - sets The document collection ID is an Integer that uniquely identifies a particular document collection.

---


### getDocumentSize

```
public int getDocumentSize()
```

:   getter for documentSize - gets The size of a document is logged as the number of characters it contains.

---


### setDocumentSize

```
public void setDocumentSize(int v)
```

:   setter for documentSize - sets The size of a document is logged as the number of characters it contains.

---


### getSecondaryDocumentIDs

```
public org.apache.uima.jcas.cas.StringArray getSecondaryDocumentIDs()
```

:   getter for secondaryDocumentIDs - gets This StringArray is used for secondary document ID storage. For example, in the biomedical domain, a particular document might be associated with a PubMed ID, however it might also have a deprecated Medline ID, or perhaps a PubMed Central ID, either of which could be stored in this StringArray. It is recommended that the type of ID along with the ID itself be stored, e.g. "MedlineID:12345".

---


### setSecondaryDocumentIDs

```
public void setSecondaryDocumentIDs(org.apache.uima.jcas.cas.StringArray v)
```

:   setter for secondaryDocumentIDs - sets This StringArray is used for secondary document ID storage. For example, in the biomedical domain, a particular document might be associated with a PubMed ID, however it might also have a deprecated Medline ID, or perhaps a PubMed Central ID, either of which could be stored in this StringArray. It is recommended that the type of ID along with the ID itself be stored, e.g. "MedlineID:12345".

---


### getSecondaryDocumentIDs

```
public String getSecondaryDocumentIDs(int i)
```

:   indexed getter for secondaryDocumentIDs - gets an indexed value - This StringArray is used for secondary document ID storage. For example, in the biomedical domain, a particular document might be associated with a PubMed ID, however it might also have a deprecated Medline ID, or perhaps a PubMed Central ID, either of which could be stored in this StringArray. It is recommended that the type of ID along with the ID itself be stored, e.g. "MedlineID:12345".

---


### setSecondaryDocumentIDs

```
public void setSecondaryDocumentIDs(int i,
                                    String v)
```

:   indexed setter for secondaryDocumentIDs - sets an indexed value - This StringArray is used for secondary document ID storage. For example, in the biomedical domain, a particular document might be associated with a PubMed ID, however it might also have a deprecated Medline ID, or perhaps a PubMed Central ID, either of which could be stored in this StringArray. It is recommended that the type of ID along with the ID itself be stored, e.g. "MedlineID:12345".

---


### getClassificationType

```
public String getClassificationType()
```

:   getter for classificationType - gets This String provides a means for classifying a particular document.

---


### setClassificationType

```
public void setClassificationType(String v)
```

:   setter for classificationType - sets This String provides a means for classifying a particular document.


---


|  |  |  |
| --- | --- | --- |
| **Overview** \| **Package** \| **Class** \| **Tree** \| **Deprecated** \| **Index** \| **Help** | |  |
| **PREV CLASS**   **NEXT CLASS** | **FRAMES**    **NO FRAMES**     **All Classes** |  |
| SUMMARY: NESTED \| FIELD \| CONSTR \| METHOD | DETAIL: FIELD \| CONSTR \| METHOD |  |


---
